# A prediction model for high ovarian response in the GnRH antagonist protocol

**DOI:** 10.3389/fendo.2023.1238092

**Published:** 2023-11-15

**Authors:** Yilin Jiang, Chenchen Cui, Jiayu Guo, Ting Wang, Cuilian Zhang

**Affiliations:** ^1^ Reproductive Medical Center, Zhengzhou University People’s Hospital, Zhengzhou, China; ^2^ Reproductive Medical Center, Henan Provincial People’s Hospital, Zhengzhou, China; ^3^ Reproductive Medical Center, Henan University People’s Hospital, Zhengzhou, China

**Keywords:** GnRH antagonist protocol, prediction model, high ovarian response, controlled ovarian stimulation, nomogram

## Abstract

**Backgrounds:**

The present study was designed to establish and validate a prediction model for high ovarian response (HOR) in the GnRH antagonist protocol.

**Methods:**

In this retrospective study, the data of 4160 cycles were analyzed following the *in vitro* fertilization (IVF) at our reproductive medical center from June 2018 to May 2022. The cycles were divided into a training cohort (n=3121) and a validation cohort (n=1039) using a random sampling method. Univariate and multivariate logistic regression analyses were used to screen out the risk factors for HOR, and the nomogram was established based on the regression coefficient of the relevant variables. The area under the receiver operating characteristic curve (AUC), the calibration curve, and the decision curve analysis were used to evaluate the performance of the prediction model.

**Results:**

Multivariate logistic regression analysis revealed that age, body mass index (BMI), follicle-stimulating hormone (FSH), antral follicle count (AFC), and anti-mullerian hormone (AMH) were independent risk factors for HOR (all *P*< 0.05). The prediction model for HOR was constructed based on these factors. The AUC of the training cohort was 0.884 (95% CI: 0.869–0.899), and the AUC of the validation cohort was 0.884 (95% CI:0.863–0.905).

**Conclusion:**

The prediction model can predict the probability of high ovarian response prior to IVF treatment, enabling clinicians to better predict the risk of HOR and guide treatment strategies.

## 1 Introduction

Infertility is a major global health problem that threatens female reproductive health. Assisted reproductive technology (ART) is considered one of the most effective treatments for infertility (1). Ovarian hyperstimulation syndrome (OHSS) is a common and severe iatrogenic complication of *in vitro* fertilization (IVF) in which clinical symptoms are ovarian enlargement, abnormal capillary permeability, excessive estradiol, ascites, and pleural effusion (2). The incidence of mild OHSS is 20%–33%, and the incidence of moderate to severe OHSS is 3%–8% (3). In addition to causing miscarriage, prolonged time to pregnancy, and pregnancy complications, severe OHSS can even cause acute renal insufficiency, acute respiratory distress, venous thrombosis, and even death of patients, which brings both economic and psychological burdens to patients (4). The main risk factor for OHSS is high ovarian response (HOR) which is defined as an abnormal sensitivity to exogenous gonadotropin, resulting in multiple follicle recruitment, development, and steroid abnormalities (5, 6).

GnRH antagonist protocol is gaining popularity among fertility centers worldwide because of its simplified procedures, reduced drug dosages, shorter treatment cycles, safety, and efficacy (7). Several studies have shown that the GnRH antagonist protocol is beneficial for HOR patients, as it can significantly reduce the risk of OHSS (8, 9). However, OHSS can’t be avoided completely, so it is essential to predict HOR to reduced it whenever possible.

Age, anti-mullerian hormone (AMH), antral follicle count (AFC), and basal sex hormones are commonly used to predict ovarian response (10), but most studies predict the risk of HOR with only one or two factors. To our knowledge, only a small amount of literatures construct the multi-variable models without the calibration curve and the decision curve analysis. In our study, we identified the independent risk factors affecting HOR and constructed a nomogram to predict the occurrence of HOR based on the results of the multivariate logistic regression. In addition, we drew the calibration curve and the decision curve to assure the accuracy and utility of the model. We aimed to create a practical algorithm to assist clinicians in implementing personalized reproductive strategies.

## 2 Materials and methods

### 2.1 Patient population and study design

This study was a single-center retrospective observational study of women who underwent IVF at the Reproductive Medicine Center of Henan Provincial People’s Hospital from June 2018 to May 2022. Enrolled as subjects of the study were patients with the GnRH antagonist protocol. The exclusion criteria were as follows: 1) cycles with missingness or outliers; 2) endocrine disorders such as abnormal thyroid function, diabetes and hyperprolactinemia; 3) tuberculosis of the reproductive system, pituitary tumors and other systemic diseases; 4) women who received preimplantation genetic testing, and those with chromosomal abnormalities; 5) canceled oocyte retrieval cycles; 6) <4 oocytes retrieved. Cycles were divided into the high ovarian response group (>15 oocytes retrieved) and the normal ovarian response group (4–15 oocytes retrieved) (11–13). The study was approved by the Ethics Committee of Reproductive Medicine of Henan Provincial People’s Hospital with the number SYSZ-LL-2021091501.

### 2.2 Ovarian stimulation

All patients adhered to the GnRH antagonist protocol. In this protocol, ovarian stimulation was initiated from day 2 or 3 of menstruation with the appropriate amount of gonadotropin at a dose of 75–300 IU/day until the hCG trigger day. During the stimulation process, the gonadotropin dose was adjusted according to follicular development, as determined by ultrasound and serum hormone levels, up to 300 IU/day. A daily dose of 0.25 mg GnRH antagonist was initiated when a dominant follicle reached a mean diameter of 12–14 mm or when the blood luteinizing hormone (LH) levels exhibited a significant upward trend until the day of hCG injection.

If there were three follicles of ≥ 16 mm diameter, two follicles of ≥ 17 mm diameter, or one follicle of ≥18 mm diameter, 5,000–10,000 IU of hCG was injected. Approximately 36–38 hours after the trigger, ultrasound-guided vaginal follicular aspiration was performed for oocyte retrieval.

### 2.3 Statistical analysis

Categorical variables included the type of infertility. Continuous variables included age, body mass index (BMI), duration of infertility, LH (luteinizing hormone), estradiol (E2), progesterone (P), follicle-stimulating hormone (FSH), antral follicle count (AFC), and anti-mullerian hormone (AMH). AFC was measured using transvaginal ultrasonography on day 2 or 3 of the menstrual cycle. FSH, LH, E2, and P were detected in the blood on day 2 or 3 of the menstrual cycle.

Normally distributed variables are presented as mean ± standard deviation (SD), and a t-test was used to compare groups. Non-normally distributed variables are presented as median (interquartile range), and the Mann-Whitney test was used for comparison. Categorical variables are presented as frequencies and percentages, and the chi-squared test was used for comparison.

Cycles with complete records were divided into training and validation cohorts using a 3:1 random sampling method. Univariate logistic regression analysis was performed in the training cohort to determine the risk factors for the occurrence of HOR. Variables with *P*< 0.05 in the univariate analysis and variables reported to be associated with HOR were included in the multivariate analysis. Variables were selected using stepwise regression and fitted to a more parsimonious model. A nomogram was established according to the regression coefficient of the relevant variables. The multicollinearity among the factors was assessed using the variance inflation factor (VIF).

The prediction performance was verified through ROC curves, and an area under the ROC curve (AUC) > 0.8 indicates good compliance with the prediction model (14). Calibration curves were used to assess the performance of the prediction model. Decision curve analysis was also performed to assess the clinical applicability of the model. Statistical analysis was performed using software packages R (http://www.R-project.org, The R Foundation) and Empower (R) (www.empowerstats.com; X&Y Solutions, Inc., Boston, MA). A two-tailed P value< 0.05 was considered statistically significant.

## 3 Results

### 3.1 Description of the study population

**Figure 1:**
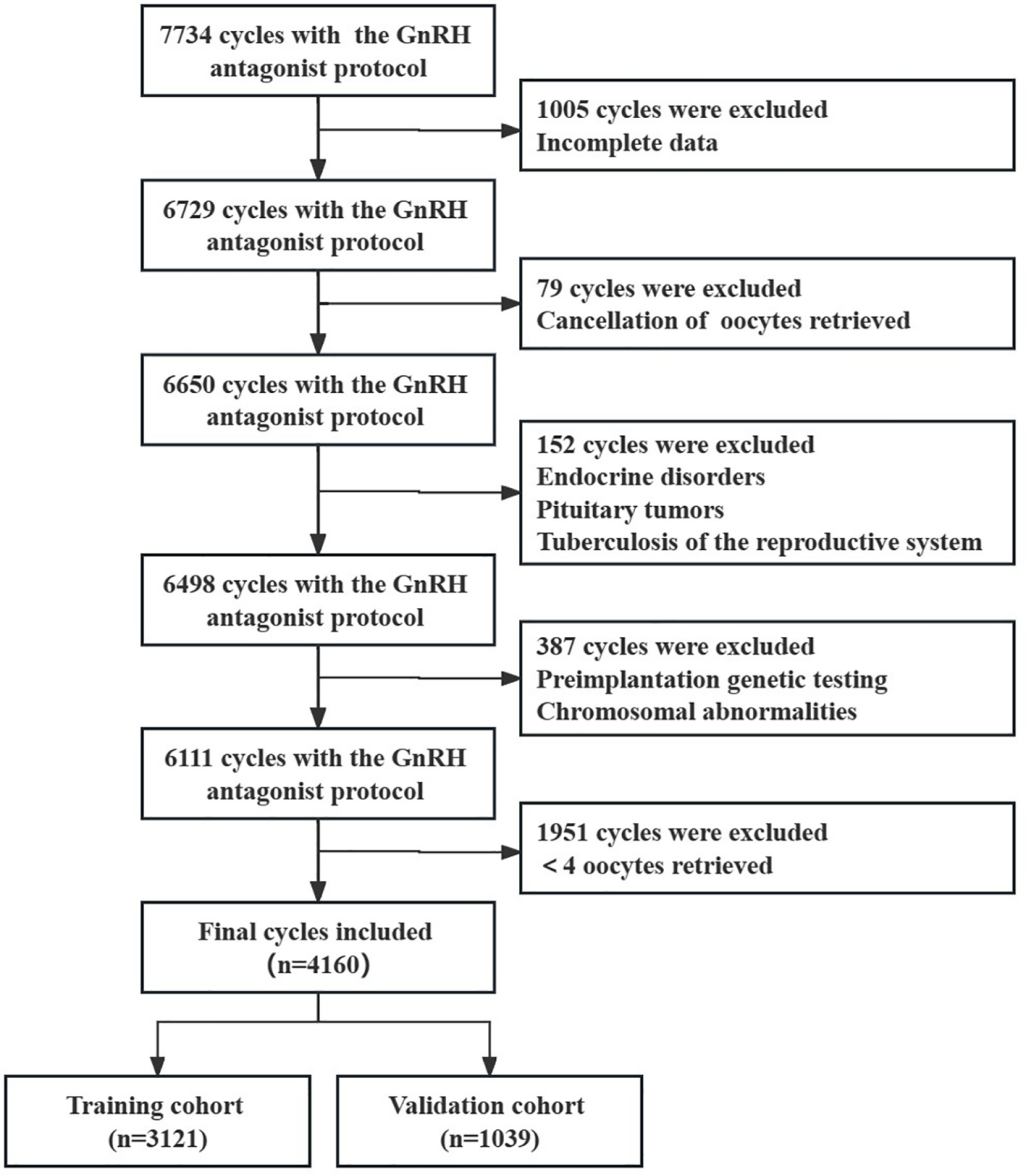
A flow-chart of cycles’ selection and exclusions.

Based on the inclusion and exclusion criteria, 4160 cycles were enrolled, with 3121 cases in the training cohort and 1039 cases in the validation cohort (**Figure 1**). The baseline characteristics of the two groups are shown in **Table 1**. There were no significant differences between the items in two groups (all *P* > 0.05).

**Table 1:** Baseline characteristics of all cycles in the training and validation cohorts.

Variables	Training cohort (n=3121)	Validation cohort (n=1039)	*Z/χ2* value	*P* value
Age (years)	33 (30,37)	33 (30,37)	0.354	0.723
BMI (kg/m^2^)	22.86 (20.80,25.39)	23.00 (20.96,25.39)	1.073	0.283
Duration of infertility (years)	3 (1.5,5)	3 (1.5,5)	0.414	0.679
Infertility type (%)			0.186	0.666
Primary	39.38 (1229/3121)	40.14 (417/1039)		
Secondary	60.62 (1892/3121)	59.87 (622/1039)		
Basal FSH (IU/L)	6.71 (5.50,8.18)	6.75 (5.65,8.28)	1.601	0.109
Basal LH (IU/L)	4.67 (3.38,6.22)	4.55 (3.44,6.19)	-0.038	0.970
Basal E2 (pg/mL)	38.33 (29.16,50.65)	37.83 (28.41,50.15)	-0.877	0.380
Basal P (ng/mL)	0.25 (0.15,0.38)	0.26 (0.15,0.38)	0.615	0.539
AMH (ng/mL)	2.15 (1.12,3.98)	1.97 (1.11,3.78)	-1.067	0.286
AFC (n)	11 (6,16)	10 (6,15)	-1.111	0.266

Continuous variables are presented as the median (interquartile range) or mean ± standard deviation. Categorical variables are presented as percentages. Positive number/total number in brackets. BMI represents body mass index, FSH represents follicle-stimulating hormone, LH represents luteinizing hormone, E2 represents estradiol, P represents progesterone, AMH represents anti-mullerian hormone, and AFC represents antral follicle counting.

#### 3.1.1 Construction of the model

**Figure 2:**
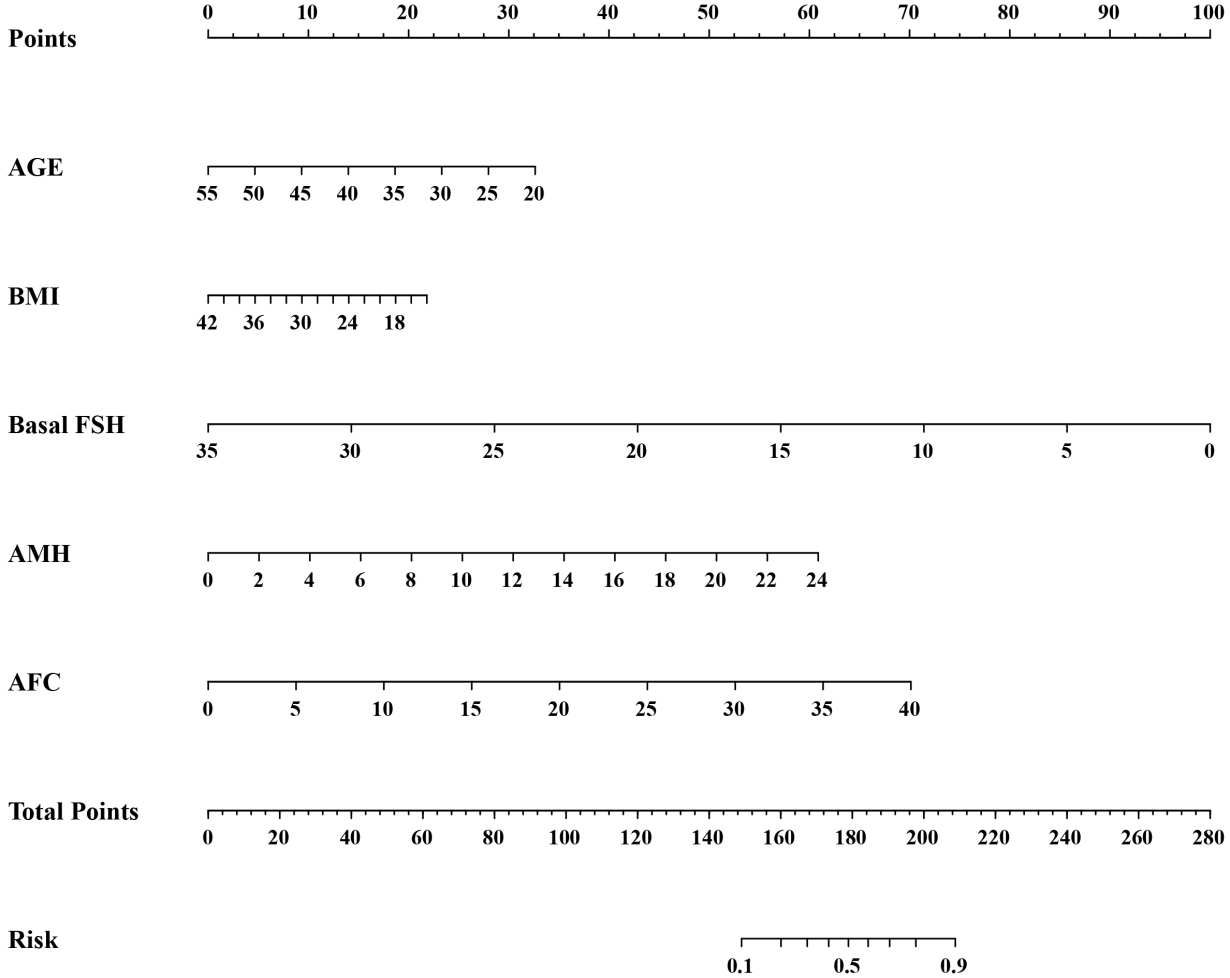
A nomogram to predict the risk of high ovarian response in the GnRH antagonist protocol.

Logistic regression analysis was used to find out the variables that included in the model. The univariate logistic regression analysis of HOR in the training cohort is listed in **Table 2**. The result suggested age, duration of infertility, type of infertility, FSH, LH, AMH, and AFC were all associated with HOR (all *P*< 0.05). Although BMI wasn’t the risk factor for HOR in our study, serval studies showed it is related to ovarian response (15). Age, duration of infertility, type of infertility, FSH, LH, AMH, AFC, and BMI were included in the multivariate analysis eventually. The multivariate logistic regression analysis revealed that AMH (OR = 1.205, 95% CI: 1.149–1.264, *P*<0.001), AFC (OR = 1.138, 95% CI: 1.108–1.168, *P*<0.001) were both risk factors for the occurrence of HOR, and age (OR = 0.934, 95% CI: 0.907–0.961, *P*<0.001), BMI (OR = 0.944, 95% CI: 0.912–0.977, *P*=0.001), FSH (OR = 0.810, 95% CI: 0.750–0.875, *P*<0.001) were all protective factors for HOR (**Table 3**). Based on the results of the multivariate logistic regression analysis, the nomogram model (**Figure 2**) was successfully established: logit (P) = 0.375-0.069 × Age-0.057 × BMI-0.210 × FSH+0.187 × AMH+0.129 × AFC. The scores of all indicators were added to obtain the total score which corresponds to the risk of HOR.

**Table 2:** Univariate analysis in the training cohort.

Variables	OR (95% CI)	*P* value
Age (years)	0.844 (0.825,0.864)	<0.001
BMI (kg/m^2^)	0.987 (0.959,1.016)	0.369
Duration of infertility (years)	0.949 (0.916,0.983)	0.003
Infertility type (%)		
Primary	Ref.	
Secondary	0.517 (0.423,0.632)	<0.001
Basal FSH (IU/L)	0.664 (0.623,0.707)	<0.001
Basal LH (IU/L)	1.141 (1.109,1.174)	<0.001
Basal E2 (pg/mL)	1.000 (0.998,1.002)	0.953
Basal P (ng/mL)	1.077 (0.923,1.256)	0.347
AMH (ng/mL)	1.520 (1.460,1.583)	<0.001
AFC (n)	1.248 (1.224,1.273)	<0.001

**Table 3:** Multivariate logistic regression model in the training cohort.

Variables	Regression coefficient	OR (95% CI)	*P* value
Age (years)	-0.069	0.934 (0.907,0.961)	<0.001
BMI (kg/m^2^)	-0.057	0.944 (0.912,0.977)	0.001
Basal FSH (IU/L)	-0.210	0.810 (0.750,0.875)	<0.001
AMH (ng/mL)	0.187	1.205 (1.149,1.264)	<0.001
AFC (n)	0.129	1.138 (1.108,1.168)	<0.001

### 3.2 Validation of the model

**Figure 3:**
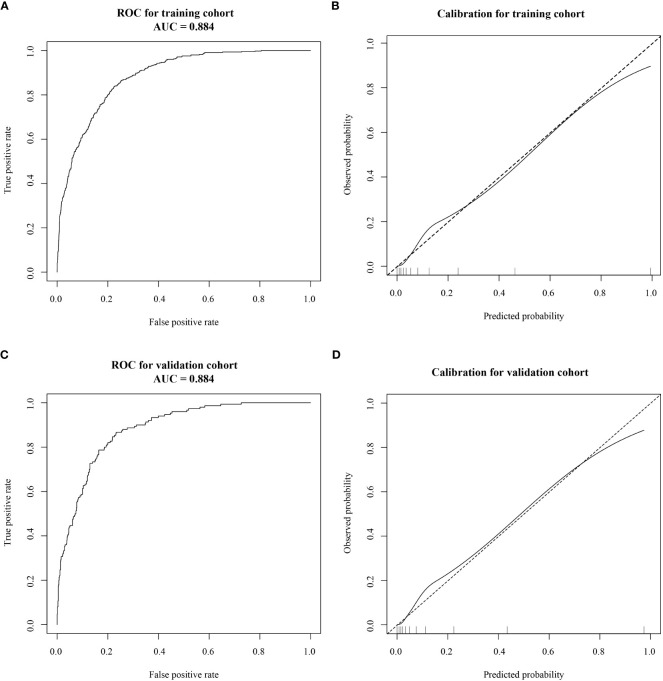
Receiver operating characteristic (ROC) curves and calibration plots of the training and validation cohorts. **(A)** The area under the ROC curve (AUC) of the training cohort was 0.884 (95% CI: 0.869–0.899). **(B)** Calibration curve for the training cohort. **(C)** The AUC of the validation cohort was 0.884 (95% CI: 0.863–0.905). **(D)** Calibration curve for the validation cohort. Calibration curves were used to evaluate the calibration of the model. The horizontal axis is the predicted probability provided by this model, and the vertical axis is the observed incidence of pregnancy failure. The ideal line with a 45° slope represents a perfect prediction (the predicted probability equals the observed probability).

**Figure 4:**
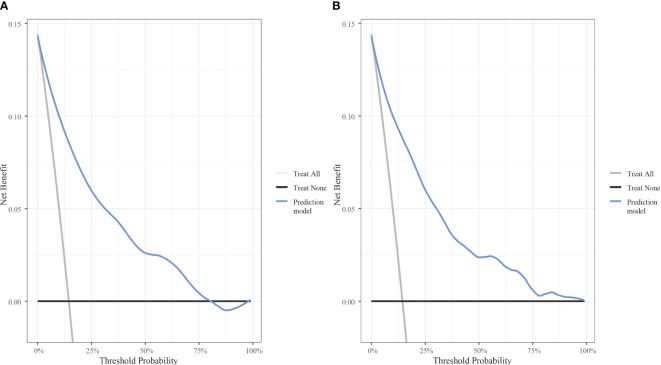
Decision curve analysis of the model with the net benefit as the vertical axis and the threshold probability as the horizontal axis. **(A)** Decision curve analysis for the training cohort. **(B)** Decision curve analysis for the validation cohort.

The AUC was calculated to verify the accuracy of the nomogram in **Figure 3**. The AUC of the training cohort was 0.884 (95% CI: 0.869–0.899), and the AUC of the validation cohort was 0.884 (95% CI: 0.863–0.905). The calibration plot revealed good predictive accuracy between actual and predicted probability in **Figure 3**. Furthermore, the decision curve analysis demonstrated that the prediction model was the higher line on the decision curve, indicating that the prediction model leads to a higher net benefit and greater clinical utility (**Figure 4**). In the training cohort, the curve demonstrated that if a patient’s threshold probability falls between 1 and 78%, utilizing the nomogram for predicting HOR offers greater benefits compared to using either the treat-all-patients or the treat-none scheme.

## 4 Discussion

Adopting individualized treatment strategies has been one of the most important topics in ART. The selection of an individualized controlled ovarian stimulation protocol is based on the ovarian response so that patients can obtain the appropriate number of follicles, embryos, and optimal pregnancy outcomes while avoiding severe adverse effects and complications, such as OHSS. Although the GnRH antagonist protocol significantly reduces the risk of OHSS, controlling and reducing it in HOR patients is a major challenge for clinicians. In our study, multivariate logistic regression analysis revealed that age, BMI, FSH, AMH, and AFC were independent risk factors for HOR in the GnRH antagonist protocol, which is consistent with the results of previous studies.

In previous studies, age and FSH often were used to assess ovarian response. Broer et al. conducted a meta-analysis showing that age was the strongest single predictor of high response (OR = 0.89, 95% CI: 0.85–0.93) (12). After the age of 35, the number of follicles decreases sharply, probably due to the incidence of chromosomal defects increases, and the sensitivity of granulosa cells to gonadotropin decreases. Some studies suggested that serum FSH increases significantly when ovarian reserve is severely declined and fluctuates significantly during the menstrual cycle; therefore, FSH has no optimal sensitivity or specificity in predicting ovarian response (16). Our data indicated that FSH was associated with ovarian response, but it shouldn’t be single-used as the predictor due to its defects.

AFC and AMH have recently become the most widely used markers for assessing ovarian reserve and ovarian response in ART. AFC is the most intuitive indicator of ovarian reserve function in patients, as it reflects the number of ovarian follicles that can be stimulated by exogenous gonadotropin in a given cycle. A review reported that AFC > 14 was a predictor of HOR (17). Another study demonstrated that the optimal threshold for predicting HOR was AFC > 16 or 18 (sensitivity: 82%, specificity: 80%) (10). However, AFC requires careful counting by skilled ultrasound operators. Of all the ovarian markers, AMH showed the best ability to predict ovarian response. AMH is mainly secreted by granulosa cells of the ovary and regulates folliculogenesis. Serum AMH reflects the number of antral follicles in the ovary, and its concentration is not affected by the menstrual cycle. Rong Li et al. demonstrated that the optimal AMH cut-off value predicting high ovarian response was 2.6 ng/mL (sensitivity: 81.28%, specificity: 59.51%) (18). Another study with 373 cycles suggested that an AMH cut-off value greater than 4.385 ng/mL may predict HOR, and the AUC was 0.845 (95% CI: 0.778–0.912) (19). Studies involving AMH have reported that the optimal threshold for predicting HOR is AMH > 3.18, 3.50, or 4.5 ng/mL (10, 20, 21). Although AMH is a reliable biomarker of ovarian response, there is no standard cut-off value because of differences in reagent kits and assays.

High BMI is associated with impaired ovarian response and has a negative effect on IVF outcomes (22). Qiu et al. noted that high BMI in PCOS patients had a negative effect on ovarian response (23). In our study, low BMI is associated with the development of HOR. BMI ≥ 28 kg/m2 is defined as obesity in China. Obesity alters the function of the hypothalamic-pituitary axis, which is associated with high levels of insulin, androgen, and estrogen and is involved in the impairment of ovarian folliculogenesis. Although obese women receive higher doses of gonadotropin and longer stimulation periods than normal weight women, they may still have poor ovarian response because of decreased drug bioavailability (24). Chalumeau et al. developed a predictive model of ovarian response, including BMI and other factors, that could explain 60% of the variance in ovarian response to stimulation (25).

Recent studies have used multi-variable models which are more credible and stable. A meta-analysis with complete available data from 1023 patients revealed that FSH, AFC, and AMH could all be combined with age for HOR prediction, where age+AMH/AFC was more accurate compared with age alone for prediction, and age+FSH exhibited a smaller increase in accuracy. The combination of age+AFC+AMH (AUC=0.85) demonstrated good accuracy for predicting HOR (12). Tan et al. reported that AFC, AMH, and P levels on the human chorionic gonadotropin (HCG) day were identified as independent predictors of HOR. The nomogram was established with the data of 480 eligible patients (26). In our study, greater number of cycles were included and a combined prediction model with age, BMI, FSH, AMH, and AFC was developed with good performance. Simultaneously, we plotted a nomogram to visualize our model. The AUC value of the combined prediction model reached 0.884, indicating the excellent discrimination of the model, and the validation confirmed the accuracy and feasibility of the model.

In the present study, risk factors of HOR were investigated and a well-calibrated prediction model was successfully proposed in patients undergoing the GnRH antagonist protocol. Our model possesses the following strengths: The variables enrolled in the nomogram can be easily measured, making it convenient to use in clinical practice. Moreover, the model was validated to ensure its reproducibility in a wider population. It was not only the discrimination and calibration but also the utility that evaluated the performance of clinical prediction models in this study. The utility of models can be assessed by Decision curve analysis (DCA), which plots net benefit (NB) at a range of clinically reasonable risk thresholds. We constructed decision models for the groups respectively, of which the results demonstrated favourable net benefits. In IVF cycles, the use of the GnRH antagonist protocol, coasting protocol, low initial gonadotropin dose, aspirin, calcium, the GnRH agonist trigger, and the whole embryo freezing strategy should be considered for patients with HOR to reduce the likelihood of OHSS, venous thrombosis, and other hazards in patients (27).

Our study also has some limitations. Environmental exposure, genetic elements, and unhealthy lifestyle habits also affect HOR, which were not included in the study because of the limited data sources. In the future, prospective, large-scale, multi-center clinical studies should be conducted to develop a more systematic and comprehensive clinical prediction model.

## 5 Conclusion

In conclusion, we aimed at infertile women using GnRH antagonist protocol in ART and successfully developed a prediction model that enables us to predict HOR patients in a simple, effective and visual way. We hope the model can help clinicians select personalized treatments to improve ART outcomes.

## Data availability statement

The raw data supporting the conclusions of this article will be made available by the authors, without undue reservation.

## Ethics statement

The studies involving humans were approved by the Ethics Committee of Reproductive Medicine of Henan Provincial People’s Hospital with the number SYSZ-LL-2021091501. The studies were conducted in accordance with the local legislation and institutional requirements. Written informed consent for participation was not required from the participants or the participants’ legal guardians/next of kin in accordance with the national legislation and institutional requirements.

## Author contributions

YJ conceived study design. TW and CC performed the data collection and statistical analysis. JG edited the manuscript. CZ was responsible for providing data and guiding study. All authors contributed to the article and approved the submitted version.
